# Subject-specific finite element modelling of the human foot complex during walking: sensitivity analysis of material properties, boundary and loading conditions

**DOI:** 10.1007/s10237-017-0978-3

**Published:** 2017-11-14

**Authors:** Mohammad Akrami, Zhihui Qian, Zhemin Zou, David Howard, Chris J Nester, Lei Ren

**Affiliations:** 1 School of Mechanical, Aerospace and Civil Engineering, University of Manchester, Manchester, M13 9PL UK; 2 Key Laboratory of Bionic Engineering, Jilin University, Changchun, 130022 People’s Republic of China; 3 School of Computing, Science and Engineering, University of Salford, Salford, M5 4WT UK; 4 Centre for Health Sciences Research, School of Health Sciences, University of Salford, Salford, M5 4WT UK

**Keywords:** Human foot, Biomechanics, Finite element analysis, Locomotion

## Abstract

The objective of this study was to develop and validate a subject-specific framework for modelling the human foot. This was achieved by integrating medical image-based finite element modelling, individualised multi-body musculoskeletal modelling and 3D gait measurements. A 3D ankle–foot finite element model comprising all major foot structures was constructed based on MRI of one individual. A multi-body musculoskeletal model and 3D gait measurements for the same subject were used to define loading and boundary conditions. Sensitivity analyses were used to investigate the effects of key modelling parameters on model predictions. Prediction errors of average and peak plantar pressures were below 10% in all ten plantar regions at five key gait events with only one exception (lateral heel, in early stance, error of 14.44%). The sensitivity analyses results suggest that predictions of peak plantar pressures are moderately sensitive to material properties, ground reaction forces and muscle forces, and significantly sensitive to foot orientation. The maximum region-specific percentage change ratios (peak stress percentage change over parameter percentage change) were 1.935–2.258 for ground reaction forces, 1.528–2.727 for plantar flexor muscles and 4.84–11.37 for foot orientations. This strongly suggests that loading and boundary conditions need to be very carefully defined based on personalised measurement data.

## Introduction

As the primary structure between the human body and the ground, the foot plays an important role during human locomotion (Alexander et al. 1987; Carrier et al. 1994; Lieberman et al. 2010). It is susceptible to damage because of the complicated and high loads experienced at the foot–ground interface and in internal tissues. Evaluation of the biomechanical factors relating to foot structure and function could be useful to better understand the aetiology of foot disorders (e.g. plantar foot ulcers), the design of physical therapies (e.g. foot orthoses) and also surgical planning (e.g. surgical implants). However, the detailed internal loading conditions, for example stress distributions within bones and soft tissues, and the contact pressures at the foot joints, are almost unmeasurable in vivo. In this scenario, computational approaches, such as finite element (FE) analysis, have already proved to be valuable in the biomechanical investigation of foot structure and function (Telfer et al. 2014).

A large number of FE models of the foot have been developed with various configurations, simplifications, material properties and loading and boundary conditions (Morales-Orcajo et al. 2016; Wang et al. 2016; Behforootan et al. 2017). The earliest models concentrated on the sagittal plane by using simplified two-dimensional (2D) geometry (Nakamura et al. 1981). With the advances in computer tomography (CT), magnetic resonance imaging (MRI) and ultrasound, three-dimensional (3D) geometries of bones and cartilages were modelled in most recent studies (Jacob et al. 1996; Gefen et al. 2000; Gefen 2002, 2003; Cheng et al. 2008; Chen et al. 2010). High-resolution CT and MRI images help reconstruct the 3D foot structure geometry of individual subjects. Using subject-specific and geometrically accurate 3D FE foot models can greatly improve our understanding of the biomechanical function of the foot during locomotion (Cheung et al. 2004, 2005, 2006).

To have clinical or industrial utility, FE foot models need to represent individual musculoskeletal structures in detail and accurately predict the adaptive behaviour of the foot in response to changes in external boundary and loading conditions. To improve model accuracy, recent studies have incorporated more structural components (based on subject-specific medical imaging data) and/or defined loading and boundary conditions based on measurement data taken on the same person (Cheng et al. 2008; Chen et al. 2010, 2012; García-González et al. 2009; Qian et al. 2010; Gu et al. 2010, 2011; Guiotto et al. 2014; Wong et al. 2015; Bae et al. 2015). However, due to the complexity of the musculoskeletal structures, most of those studies have involved simplification of some parts of the foot structure, and/or simplified loading and boundary conditions. For example, the 3D plantar fascia structure has been modelled as one-dimensional (1D) truss elements (García-González et al. 2009; Chen et al. 2010; Qian et al. 2010; Bae et al. 2015; Wong et al. 2015), and foot bones fused preventing articular motion that occurs in vivo (Guiotto et al. 2014). In many models, only vertical or sagittal plane loading and/or boundary conditions were applied (Cheng et al. 2008; Chen et al. 2010; Gu et al. 2010; García-González et al. 2009; Chen et al. 2012; Guiotto et al. 2014; Bae at al. 2015). In addition, most models used muscle forces either from literature data (Chen et al. 2010; Cheung et al. 2005; Guiotto et al. 2014; Wong et al. 2015) or based on simplified assumptions (Chen et al. 2012; Bae et al. 2015). Moreover, although most models were validated against measured plantar pressure data, the experimental validations were conducted either by comparing the distribution pattern qualitatively (Chen et al. 2010; Gu et al. 2010; Qian et al. 2010; Bae et al. 2015; Wong et al. 2015), or by comparing the peak pressures in large areas, e.g. forefoot, mid-foot and hind foot (Guiotto et al. 2014) rather than at specific anatomical sites (e.g. individual metatarsal heads).

The objective of this study was to construct and validate a subject-specific FE foot model. This was achieved by integrating medical imaging-based FE musculoskeletal modelling, multi-body musculoskeletal modelling and 3D gait measurements, all derived from the same subject. The FE model comprises of major ankle–foot musculoskeletal components, including 30 bones, 85 ligament bundles, 74 cartilage layers, 3D bulk plantar fascia, 3D solid Achilles tendon and the encapsulated soft tissue. Individualised 3D gait measurement data, and muscle force data provided by the multi-body musculoskeletal model, were used to define the subject-specific boundary and loading conditions. A region-specific experimental validation was conducted to compare predicted plantar pressure at five events during the stance phase of walking against subject-specific barefoot walking pressures. Sensitivity analysis was performed to investigate the effects of variations in material properties, loading and boundary conditions on model predictions. The capability of the FE model to predict adaptive behaviours of the foot in response to variations in ground–foot interactions, muscle loads and foot orientation was also investigated.

**Fig. 1:**
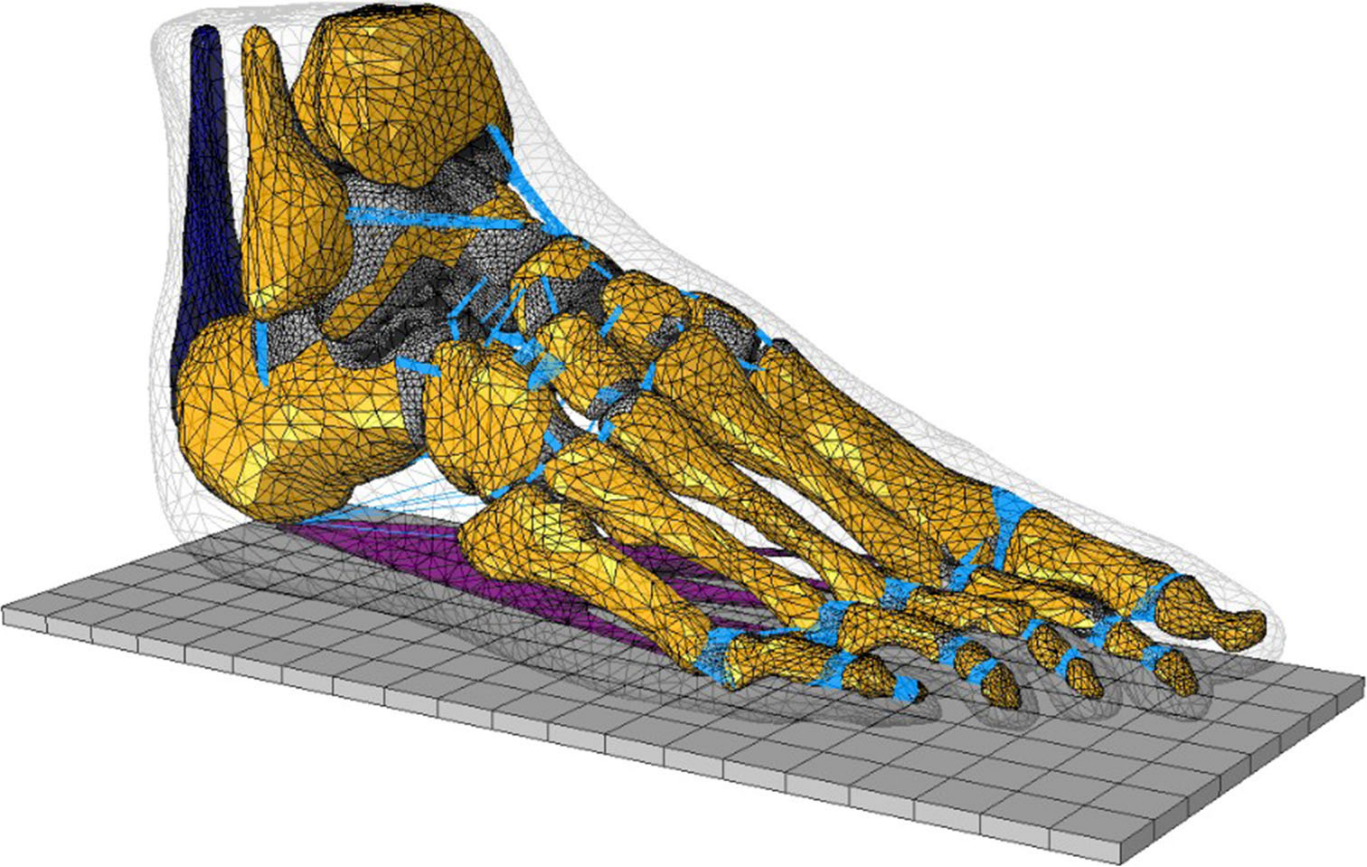
Finite element model of the foot and ankle musculoskeletal complex, including 30 bones, 85 ligament bundles with 1814 line elements, 74 cartilage layers, plantar fascia and encapsulated soft tissue (transparent)

## Materials and methods

### Ethics statement

The subject gave informed consent to participate in the MRI scanning and motion capture measurements, which were approved by the institutional review board committee.

### Finite element modelling

The 3D geometry of foot structures and the foot were reconstructed from medical MRI images (2-mm slice interval) (MAGNETOM Avanto 1.5T, Siemens AG, Germany) obtained by scanning the right foot of a healthy male subject (age: 27 years; weight: 75 kg; no history of lower limb injury or foot abnormalities). He lay with the foot approximately $90{^{\circ }}$ to the leg and loaded on a flat plastic plate. The images were segmented to obtain the boundaries of bones and soft tissues using Mimics software (Materialise, Leuven, Belgium). SolidWorks (Dassault Systèmes, SolidWorks Corp., USA) was used to process boundary surfaces and build solid bone and soft tissue models. Thirty bony structures were constructed (calcaneus, talus, cuboid, navicular, 3 cuneiforms, 5 metatarsals, 14 phalanges, medial and lateral sesamoids and the distal parts of the tibia and fibula) (see Fig. 1). Seventy-four cartilage layers were modelled for 37 pairs of articulations between the 30 bones. Surface-to-surface frictionless contact was used to represent the relative articulating movements between cartilages layers. This allows the bones to slide over one another without friction.

A total of 1814 truss elements were used to model the biomechanical constraints provided by 85 ligament bundles in the ankle–foot musculoskeletal complex (see Fig. 1). Those ligament elements were considered to have a physiological cross-sectional area (PCSA) and respond to tension only. The plantar fascia was constructed by connecting the medial calcaneal tubercle to the proximal phalanges of the toes (see Fig. 1). The Achilles tendon was incorporated into the upper ridge of the calcaneus (see Fig. 1). This allows the application of muscle forces from lateral and medial gastrocnemius (LG, MG) and soleus (SOL) by applying a uniformly distributed tension through cross-sectional area of the Achilles tendon (see the finite element simulations during walking section for details of foot muscle force application). A 3D volume of soft tissues was modelled to encapsulate all the bony and ligamentous foot musculoskeletal components (see Fig. 1).

The upper surfaces of the tibia, fibula and the encapsulated soft tissue were totally fixed. A 3D solid plate was used to simulate the ground, which was only allowed to move along the direction defined by the measured 3D GRF vector. The interaction between the foot plantar surface and the ground was defined with a frictional coefficient of 0.6 based on values for in vivo skin-ground frictional properties (Zhang and Mak 2009). The material properties of all the foot bony and ligamentous components and the ground plate were idealised as homogenous isotropic and linear elastic with different Young’s moduli and Poisson’s ratios based on literature data. The material properties and element type used for modelling different components of the foot and ground plate are listed in Table 1. The mesh was determined through a convergence analysis by gradually increasing the mesh density until the deviations in the estimated stresses reached < 5%.

**Table 1:** Material properties and element types of the finite element model

Components	Materials	Element types	Young’s modulus (MPa)	Poisson’s ratio	References
Bone	Solid, linear elastic	Tetrahedral	7300	0.3	Nakamura et al. (1981)
Cartilage	Solid, linear elastic	Tetrahedral	1	0.4	–
Ligament	Tension only	Truss	260	0.4	–
Plantar fascia	Solid, linear elastic	Tetrahedral	350	0.4	–
Achilles tendon	Solid, linear elastic	Tetrahedral	816	0.3	Chen et al. (2012)
Encapsulated soft tissue	Solid, linear elastic	Tetrahedral	1.15	0.49	–
Ground support	Solid, linear elastic	Tetrahedral	17,000	0.1	–

### Gait measurements and muscle forces estimation

**Fig. 2:**
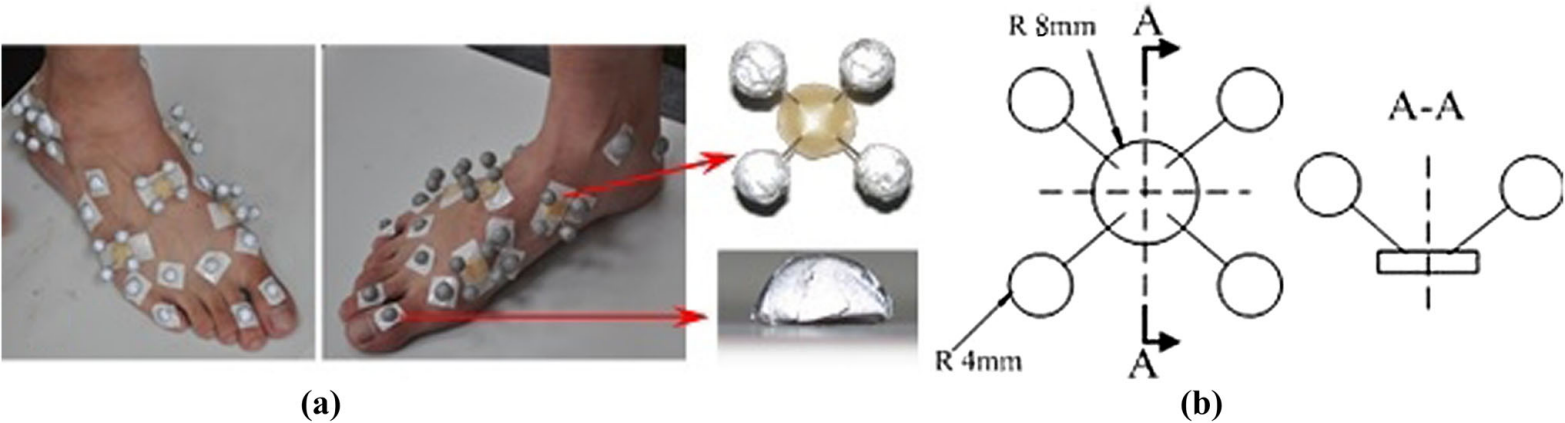
Infrared maker cluster system used in this study to capture 3D foot motions. **a** The foot was divided into five segments including hindfoot, mid-foot, medial and lateral forefoot and toes. A set of thermal plastic plates with each carrying four infrared markers were mounted firmly on each segment to capture the segmental motions. A number of hemispherical infrared markers were also attached on the anatomical landmarks. **b** The configuration of the rigid marker cluster and the hemispherical marker

Three-dimensional gait measurement was taken on the same subject used for MRI scans and FE model construction. Data were used to inform and validate the FE modelling and collected based on a previously established experimental protocol (Qian et al. 2013). A 12-camera infrared motion analysis system (Qualisys, Sweden) was used to capture the 3D motions of the trunk and lower limb segments at 150 Hz. A six force plate array (Kistler, Switzerland) was used to record the 3D ground reactions at 1000 Hz, and a 1-metre-long pressure plate (RSscan, Belgium) was used for foot pressure distribution (at 250 Hz). The camera system and force plates were digitally synchronised using a manual trigger. A set of infrared reflective marker clusters mounted on thermoplastic plates were used (same to those used in Ren et al. 2008) to capture the 3D motions of the trunk, pelvis, thighs, shanks and multi-segment foot motion (see Fig. 2). The calibrated anatomical system technique (Ren et al. 2008; Cappozzo et al. 1995) was used to determine anatomical landmarks. The subject was instructed to walk barefoot at normal walking speed along a level walkway. Ten trials were recorded to ensure a representative gait pattern was obtained. The measurement data were processed using GMAS software, a MATLAB-based software package for 3D kinematic and kinetic analysis of general biomechanical multi-body systems (Ren et al. 2008). The marker data were filtered using a low-pass zero lag fourth-order Butterworth digital filter with a cut-off frequency of 6.0 Hz.

A 3D musculoskeletal multi-body model was constructed for the same subject used in the MRI scans, FE model construction and gait measurements. This predicted leg muscle forces during walking. The model was developed based on a generic lower limb musculoskeletal model (Delp et al. 1990) available in the OpenSim software as ‘gait2392’. It consists of 92 musculotendon units to represent 76 muscles in the lower limbs and torso (Delp et al. 2007). The lower limb has seven segments: pelvis, femur, patella, tibia/fibula, talus, foot (calcaneus, navicular, cuboid, cuneiforms, metatarsals) and toes. The model was scaled based on the anatomical landmarks defined for each body segment in the gait measurements (Ren et al. 2008). The processed marker data and the recorded 3D ground reactions of a representative gait cycle (walking speed 1.58 ${\hbox {ms}}^{-1}$) were used as input to the musculoskeletal model. Thereafter, the muscle forces of all the leg muscles during walking were calculated using the static optimisation method in OpenSim software (Delp et al. 2007). The obtained muscle forces of six major ankle–foot muscles (medial and lateral gastrocnemius, soleus, tibialis posterior, peroneus longus and tibialis anterior) were used to define the muscle loading condition for FE foot simulations.

### Finite element simulations of walking

**Fig. 3:**
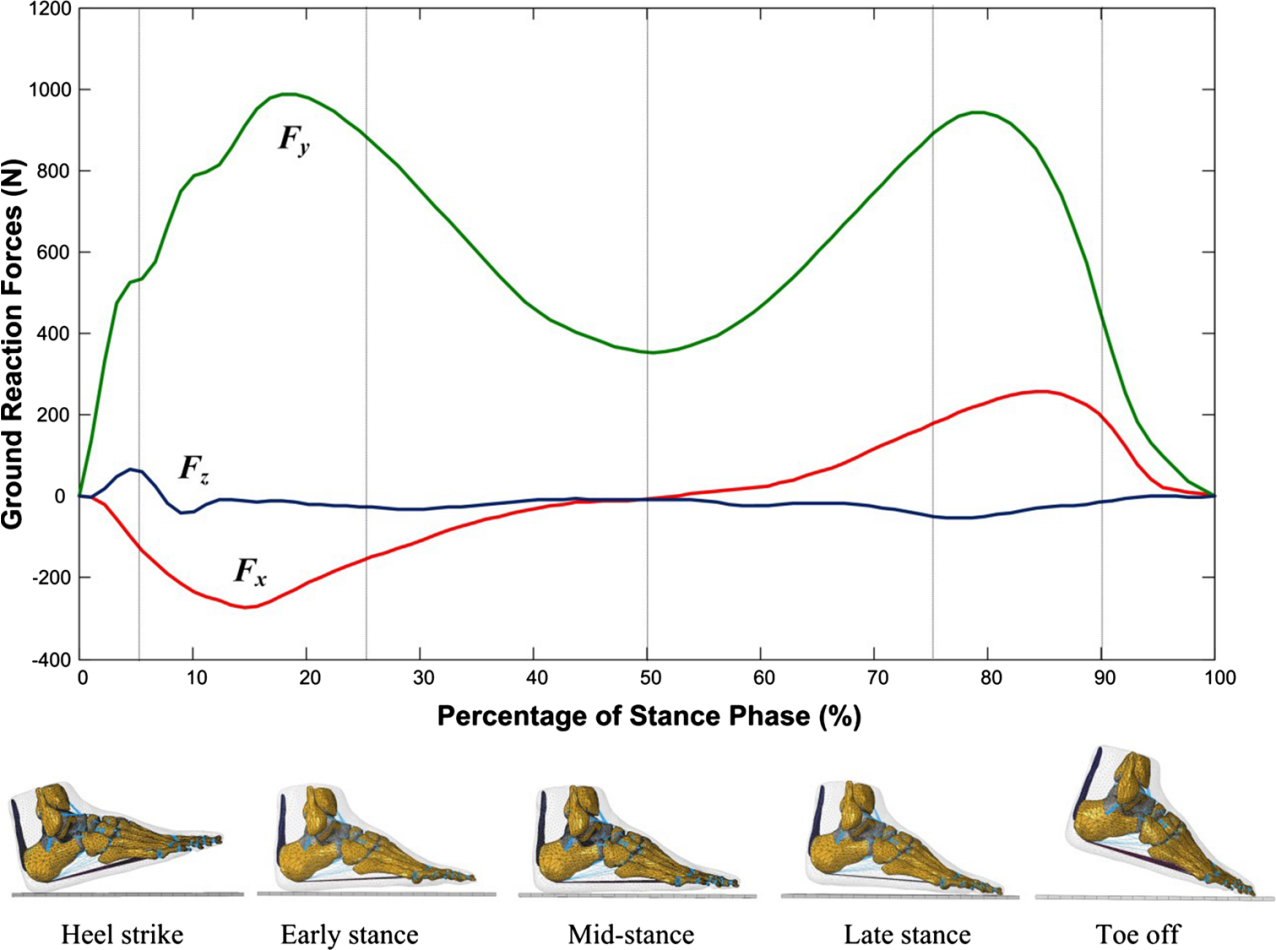
Measured 3D ground reaction forces from a representative walking trial at self-selected normal walking speed ($1.58\,\hbox {ms}^{-1})$ used to define the loading conditions of the FE simulations at five different gait instants: heel strike (at 5% of the stance phase), early stance (25% of the stance phase), mid-stance (50% of the stance phase), late stance (75% of the stance phase) and toe off (90% of the stance phase)

**Fig. 4:**
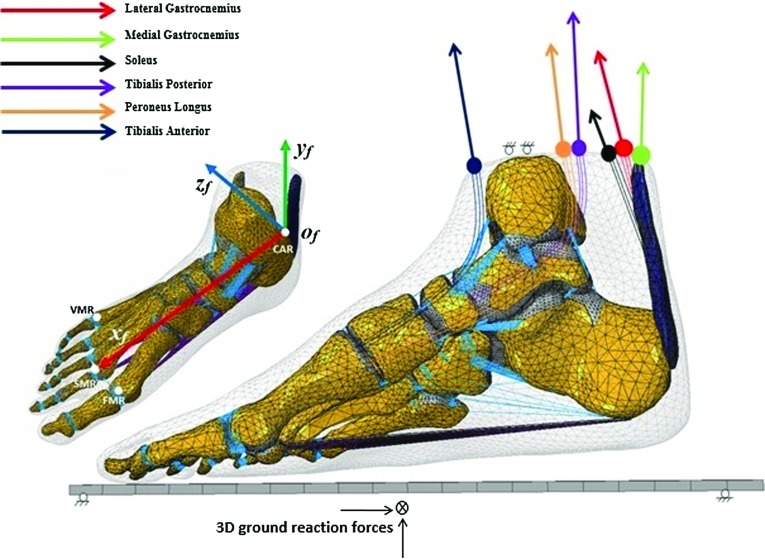
Boundary and loading conditions of the finite element foot model. The 3D orientation of the foot with respect to the ground at five different gait events was determined by the local foot coordinate system $x_{f}y_{f}z_{f}o_{f}$ defined by four anatomical landmarks CAR, FMR, SMR and VMR (Ren et al. 2008). Measured 3D ground reaction forces were applied on the ground plate and muscle forces of six major muscles (lateral gastrocnemius, medial gastrocnemius, soleus, tibialis posterior, peroneus longus, tibialis anterior) were applied at their origin/insertion attachment sites

FE foot simulations at five gait events (heel strike, early stance, mid-stance, late stance and toe off) (see Fig. 3) were performed using ABAQUS software (Simulia, Providence, USA). During each simulation, the superior surfaces of the tibia, fibula and soft tissues were fully fixed to simulate the constraints from proximal tissues. The measured 3D ground reaction forces (GRFs) $F_{x}$, $F_{y}$ and $F_{z}$ from a representative walking trial (walking speed $1.58\,\hbox {ms}^{-1}$) were applied to the ground plate at the measured centre of pressure. This 3D force application was constrained to move in the GRF vector direction only (see Fig. 4). The 3D orientation of the foot with respect to the ground at each of the five stance events was determined by the three Euler angles ($\alpha ,\beta , \gamma$) of the foot anatomical coordinate system with respect to the global coordinate system fixed on the ground during the gait measurements. The foot anatomical coordinate system was defined by four anatomical landmarks based on the previous gait measurement study ((Ren et al. 2008)) (see the inset of Fig. 4).

The six muscle forces calculated using the OpenSim model were applied to the FE foot model for each of the five gait events. The lateral and medial gastrocnemius and soleus muscle forces were applied assuming a uniform distribution of tension through the cross-sectional area of the Achilles tendon along the 3D direction of each muscle force vector (see Fig. 4). For tibialis posterior, peroneus longus and tibialis anterior muscle forces were applied at their corresponding insertion sites using a uniformly distributed muscle tension along the direction of each 3D muscle force vector determined by the OpenSim model (see Fig. 4). Table 2 lists the values of the 3D foot orientation angles ($$\alpha ,\beta ,\gamma $$), 3D GRFs ($$F_{x}$$, $$F_{y}$$ and $$F_{z})$$ and the muscle forces used to define the boundary and loading conditions for the FE simulations.

**Table 2:** Measured 3D foot orientation angles, 3D ground reaction forces and calculated muscle forces used to define the loading and boundary conditions of the finite element model at five different gait instants in the stance phase of walking of a representative trial (speed: $1.58\,\hbox {ms}^{-1}$)

Parameter	Gait events in stance phase of walking				
	Heel strike	Early stance	Mid-stance	Late stance	Toe off
*3D foot orientation angles (degree)*					
Alpha $(\alpha )$	19.15	10.36	9.71	13.08	19.20
Beta $(\beta )$	178.55	178.80	178.09	175.93	167.01
Gamma $(\gamma )$	7.08	$-$ 6.78	$-$ 7.15	$-$ 17.14	$-$ 49.35
*3D ground reaction forces (N)*					
Anterior $(F_{x})$	$-$ 95.17	$-$ 152.01	$-$ 13.11	150.87	192.33
Vertical $(F_{y})$	506.52	879.85	388.15	869.55	474.64
Lateral $(F_{z})$	59.91	$-$ 41.03	$-$ 4.63	$-$ 45.68	$-$ 18.79
*Muscle forces (N)*					
Medial gastrocnemius	79.97	15.51	592.35	1104.75	47.46
Lateral gastrocnemius	13.31	7.36	145.82	240.43	11.58
Soleus	21.57	193.62	776.79	1179.61	963.07
Tibialis posterior	18.30	638.94	572.56	383.35	295.55
Tibailis anterior	154.23	107.35	79.00	36.32	9.81
Peroneus longus	82.78	9.50	9.05	11.10	13.43

### Model validation and sensitivity analysis

**Fig. 5:**
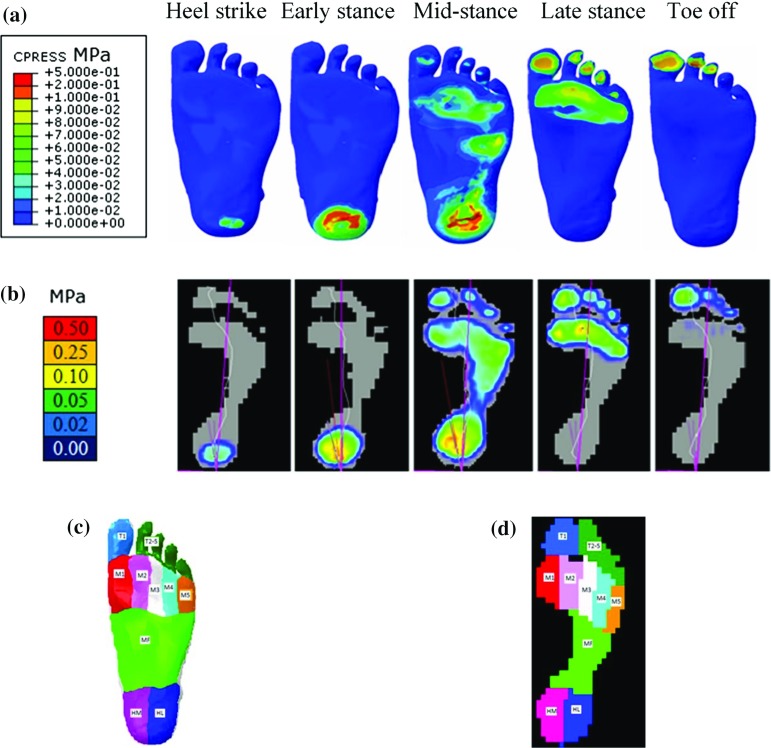
FE simulated plantar pressure distribution (**a**) compared to the recorded pressure plate data (**b**) during normal walking at five different gait events: heel strike, early stance, mid-stance, late stance and toe off. The peak and average plantar pressures were analysed in this study at ten plantar regions (T1, T2–5, M1, M2, M3, M4, M5, MF, HM, HL) of the foot model (**c**) and also the measured pressure plate data (**d**). (T1: toe 1, T2–5: toe 2–5, M1: meta 1, M2: meta 2, M3: meta 3, M4: meta 4, M5: meta 5, MF: mid-foot, HM: heel-medial, HL: heel-lateral)

To validate the FE foot model, the simulated plantar pressures at each of the five gait events were compared to the corresponding pressure plate data measured for the same subject during barefoot walking. The foot plantar area was divided into ten regions for both the FE foot model (see Fig. 5c) and the pressure plate data (see Fig. 5d) (heel-medial, heel-lateral, mid-foot, each of the 5 metatarsals, toe 1, toes 2–5). The predicted region-specific peak and average plantar pressures were validated against the corresponding pressure plate data for all the ten plantar regions at all five gait events.

**Fig. 6:**
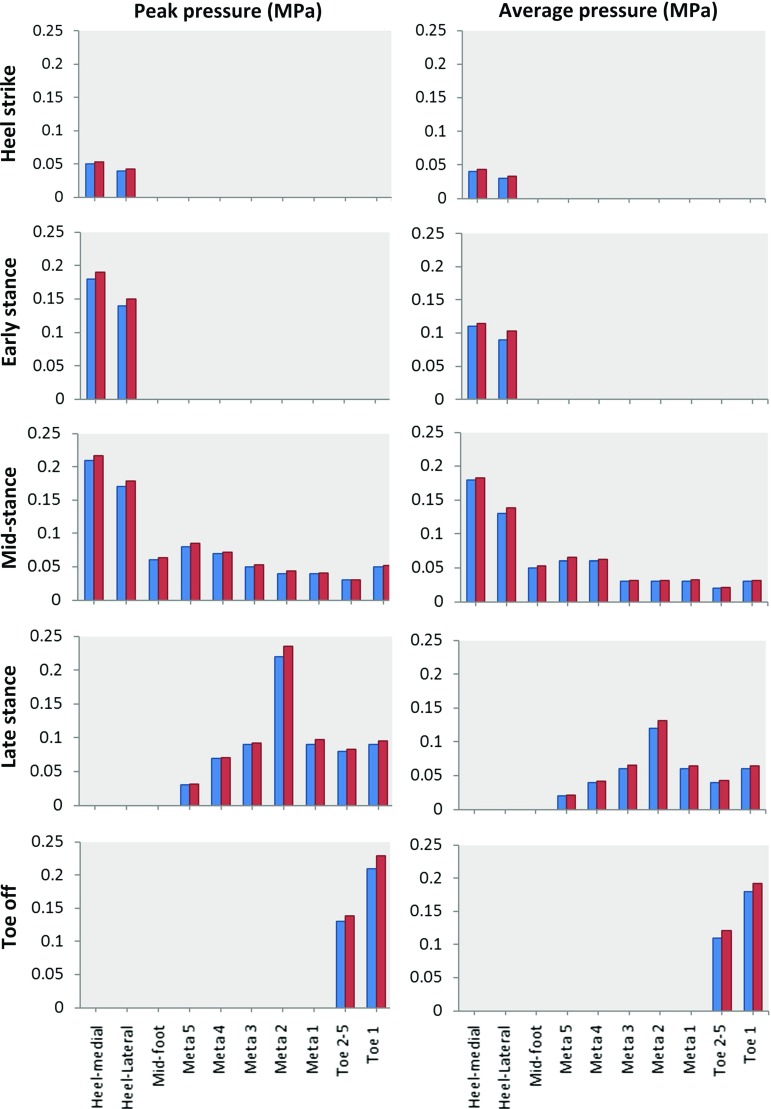
Simulated peak and average plantar pressures (red) compared to the measured peak and average pressure plate data (blue) in ten plantar regions at five different gait instants (heel strike, early stance, mid-stance, late stance, toe off) of a representative normal walking trial (speed: $1.58\,\hbox {ms}^{-1})$

A series of sensitivity analyses were conducted to investigate the effect of material properties, 3D foot orientation, 3D GRFs and muscle forces on the model predictions of plantar pressure in the mid-stance of walking. In the material property analysis, Young’s modulus (*E*) of the encapsulated soft tissue was altered by $+$ 20, $+$ 10, $-$ 10 and $-$ 20% from the baseline (1.15 MPa). For the 3D foot angle sensitivity analysis, there were eight cases in which the two major foot orientation angles $\alpha$ and $\gamma$ were changed by $+$ 2, $+$ 1, $-$ 1 and $-$ 2% from their baseline values ($9.70{^{\circ }}$ and $-\,7.15{^{\circ }}$, respectively). The 3D GRF analysis included eight cases in which the two dominant GRF components $F_{x}$ and $F_{y}$ were changed by $+$ 10, $+$ 5, $-$ 5 and $-$ 10% from their baseline values (13.11 and 145.82 N, respectively). The muscle force analysis included twelve cases in which LG, MG and SOL muscle forces were changed by $+$ 10, $+$ 5, $-$ 5 and $-$ 10% from their baseline values (145.82, 592.35 and 776.79 N, respectively).

**Table 3:** Simulated peak and average plantar pressures compared to the pressure plate data in ten plantar regions at five different gait instants in the stance phase of normal walking (speed: $1.58\,\hbox {ms}^{-1})$

Gait events	Plantar region	Peak plantar pressure (MPa)			Average plantar pressure (MPa)		
		Measurement	Simulated	Error (%)	Measurement	Simulated	Error (%)
*Heel strike*	Heel-medial	0.05	0.053	$-$ 6.00	0.04	0.043	$-$ 7.50
	Heel-lateral	0.04	0.042	$-$ 5.00	0.03	0.033	$-$ 10.00
	Mid-foot	0	0	0	0	0	0
	Meta 5	0	0	0	0	0	0
	Meta 4	0	0	0	0	0	0
	Meta 3	0	0	0	0	0	0
	Meta 2	0	0	0	0	0	0
	Meta 1	0	0	0	0	0	0
	Toe 2–5	0	0	0	0	0	0
	Toe 1	0	0	0	0	0	0
*Early stance*	Heel-medial	0.18	0.190	$-$ 5.55	0.11	0.114	$-$ 3.64
	Heel-lateral	0.14	0.150	$-$ 7.14	0.09	0.103	$-$ 14.44
	Mid-foot	0	0	0	0	0	0
	Meta 5	0	0	0	0	0	0
	Meta 4	0	0	0	0	0	0
	Meta 3	0	0	0	0	0	0
	Meta 2	0	0	0	0	0	0
	Meta 1	0	0	0	0	0	0
	Toe 2–5	0	0	0	0	0	0
	Toe 1	0	0	0	0	0	0
*Mid-stance*	Heel-medial	0.21	0.217	$-$ 3.33	0.18	0.183	$-$ 1.67
	Heel-lateral	0.17	0.179	$-$ 5.29	0.13	0.138	$-$ 6.15
	Mid-foot	0.06	0.063	$-$ 5.00	0.05	0.053	$-$ 6.00
	Meta 5	0.08	0.085	$-$ 6.25	0.06	0.065	$-$ 8.33
	Meta 4	0.07	0.072	$-$ 2.86	0.06	0.062	$-$ 3.33
	Meta 3	0.05	0.053	$-$ 6.00	0.03	0.032	$-$ 6.67
	Meta 2	0.04	0.044	$-$ 10.00	0.03	0.032	$-$ 6.67
	Meta 1	0.04	0.041	$-$ 2.50	0.03	0.033	$-$ 10.00
	Toe 2–5	0.03	0.031	$-$ 3.33	0.02	0.021	$-$ 5.00
	Toe 1	0.05	0.052	$-$ 4.00	0.03	0.032	$-$ 6.67
*Late stance*	Heel-medial	0	0	0	0	0	0
	Heel-lateral	0	0	0	0	0	0
	Mid-foot	0	0	0	0	0	0
	Meta 5	0.03	0.032	$-$ 6.67	0.02	0.021	$-$ 5.00
	Meta 4	0.07	0.071	$-$ 1.43	0.04	0.042	$-$ 5.00
	Meta 3	0.09	0.092	$-$ 2.22	0.06	0.065	$-$ 8.33
	Meta 2	0.22	0.235	$-$ 6.82	0.12	0.131	$-$ 9.17
	Meta 1	0.09	0.097	$-$ 7.78	0.06	0.064	$-$ 6.67
	Toe 2–5	0.08	0.083	$-$ 3.75	0.04	0.043	$-$ 7.50
	Toe 1	0.09	0.095	$-$ 5.56	0.06	0.064	$-$ 6.67
*Toe off*	Heel-medial	0	0	0	0	0	0
	Heel-lateral	0	0	0	0	0	0
	Mid-foot	0	0	0	0	0	0
	Meta 5	0	0	0	0	0	0
	Meta 4	0	0	0	0	0	0
	Meta 3	0	0	0	0	0	0
	Meta 2	0	0	0	0	0	0
	Meta 1	0	0	0	0	0	0
	Toe 2–5	0.13	0.138	$-$ 6.15	0.11	0.121	$-$ 10.00
	Toe 1	0.21	0.229	$-$ 9.05	0.18	0.192	$-$ 6.67

**Table 4:** Simulated peak and average plantar pressures (MPa) and their percentage changes (%) with respect to (w.r.t.) the baseline values in response to the change in Young’s modulus of the encapsulated soft tissue in ten plantar regions

Plantar region	Young’s modulus of encapsulated soft tissue in MPa (percentage change w.r.t. baseline)				
	0.92 ($-$ 20%)	1.035 ($-$ 10%)	1.15 (baseline)	1.265 ($+$ 10%)	1.38 ($+$ 20%)
*Peak plantar pressure in MPa (percentage change % w.r.t. baseline)*					
Heel-medial	0.188 ($-$ 13.3)	0.199 ($-$ 8.3)	0.217	0.232 ($+$ 6.9)	0.271 ($+$ 24.9)
Heel-lateral	0.164 ($-$ 8.3)	0.173 ($-$ 3.4)	0.179	0.193 ($+$ 7.8)	0.222 ($+$ 24.0)
Mid-foot	0.058 ($-$ 7.9)	0.060 ($-$ 4.8)	0.063	0.072 ($+$ 14.3)	0.098 ($+$ 55.6)
Meta 5	0.067 ($-$ 21.2)	0.078 ($-$ 8.2)	0.085	0.089 ($+$ 4.7)	0.096 ($+$ 12.9)
Meta 4	0.057 ($-$ 20.8)	0.064 ($-$ 11.1)	0.072	0.077 ($+$ 6.9)	0.084 ($+$ 16.7)
Meta 3	0.041 ($-$ 22.6)	0.045 ($-$ 15.1)	0.053	0.061 ($+$ 15.1)	0.067 ($+$ 26.4)
Meta 2	0.037 ($-$ 15.9)	0.041 ($-$ 6.8)	0.044	0.051 ($+$ 15.9)	0.062 ($+$ 40.9)
Meta 1	0.035 ($-$ 14.6)	0.039 ($-$ 4.9)	0.041	0.044 ($+$ 7.3)	0.048 ($+$ 17.1)
Toes 2–5	0.026 ($-$ 16.1)	0.029 ($-$ 6.5)	0.031	0.033 ($+$ 6.5)	0.041 ($+$ 32.3)
Toe 1	0.039 ($-$ 25.0)	0.046 ($-$ 11.5)	0.052	0.059 ($+$ 13.5)	0.063 ($+$ 21.2)
*Average plantar pressure in MPa (percentage change % w.r.t. baseline)*					
Heel-medial	0.154 ($-$ 15.9)	0.168 ($-$ 8.2)	0.183	0.198 ($+$ 8.2)	0.215 ($+$ 17.5)
Heel-lateral	0.117 ($-$ 15.2)	0.127 ($-$ 8.0)	0.138	0.150 ($+$ 8.7)	0.163 ($+$ 18.1)
Mid-foot	0.041 ($-$ 22.6)	0.048 ($-$ 9.4)	0.053	0.059 ($+$ 11.3)	0.067 ($+$ 26.4)
Meta 5	0.058 ($-$ 10.8)	0.061 ($-$ 6.2)	0.065	0.070 ($+$ 7.7)	0.076 ($+$ 16.9)
Meta 4	0.052 ($-$ 16.1)	0.057 ($-$ 8.1)	0.062	0.068 ($+$ 9.7)	0.074 ($+$ 19.4)
Meta 3	0.025 ($-$ 21.9)	0.029 ($-$ 9.4)	0.032	0.037 ($+$ 15.6)	0.042 ($+$ 31.5)
Meta 2	0.024 ($-$ 25.0)	0.028 ($-$ 12.5)	0.032	0.035 ($+$ 9.4)	0.039 ($+$ 21.9)
Meta 1	0.026 ($-$ 21.2)	0.030 ($-$ 9.1)	0.033	0.035 ($+$ 6.1)	0.040 ($+$ 21.2)
Toes 2–5	0.015 ($-$ 28.6)	0.019 ($-$ 9.5)	0.021	0.024 ($+$ 14.3)	0.026 ($+$ 23.8)
Toe 1	0.025 ($-$ 21.9)	0.029 ($-$ 9.4)	0.032	0.035 ($+$ 9.4)	0.039 ($+$ 21.9)

## Results

Figure 5a, b shows the predicted plantar pressure distributions compared to the corresponding measured plantar pressure data at the five stance events. The location with the highest plantar pressure moved from the heel region to the toes over the stance phase, which was consistent with the measured pressure plate data. Figure 6 shows the predicted peak and average plantar pressures in the ten plantar regions at the five gait events compared to the measured plantar pressure data. Predicted and measured peak and average pressure data and the percentage errors are given in Table 3. All the peak and average pressure percentage errors were below 10% in all the ten plantar regions for all the five gait events with only one exception ($-$ 14.44% for the average pressure in the heel-lateral region at the early stance). The maximum peak pressure percentage error was 7.14% for the heel-medial and heel-lateral regions, 5% for the mid-foot region, 10% for the meta 1 and meta 2 regions, 6.67% for the meta 3, meta 4 and meta 5 regions, and 9.05% for the toe 1 and toe 2–5 regions for all five gait events.

Table 4 contains results of the sensitivity analysis for the encapsulated soft tissue material properties. The plantar pressures increased with hardened soft tissue and decreased with softened soft tissue. The percentage changes in the peak and average plantar pressures in all the ten regions largely vary linearly with changing Young’s modulus. The peak and average plantar pressures in the metatarsal 2 and 3, toes 2–5 and mid-foot regions were more sensitive to tissue hardening with disproportionately increased pressures when the Young’s modulus increased. The maximum region-specific percentage change ratio (peak stress percentage change over parameter percentage change) is 1.245 for the heel-medial and heel-lateral regions, 2.78 for the mid-foot region, 2.045 for meta 1 and meta 2 regions, 1.51 for the meta 3, meta 4 and meta 5 regions and 1.615 for toe 1 and toe 2–5 regions. Outcomes are thus region specific.

**Fig. 7:**
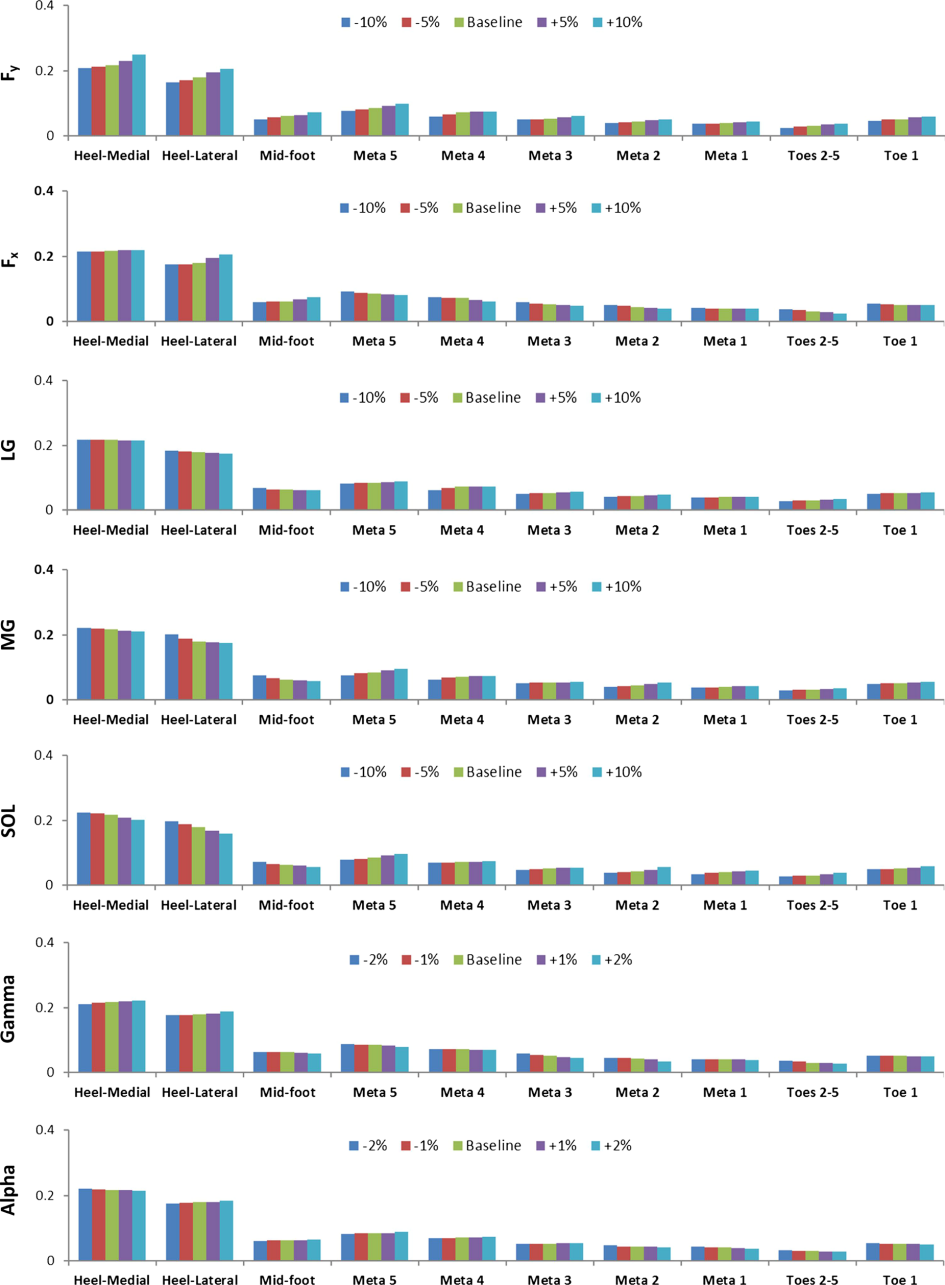
Sensitivity analysis results of peak plantar pressures (in MPa) at ten plantar regions in the mid-stance of walking by varying ground reaction forces $F_{x}$ and $F_{y}$, muscle forces of medial and lateral gastrocnemius and soleus, and also the foot orientation angles $\alpha$ and $\gamma$

Figure 7 shows the sensitivity analysis results for variations in GRF $F_{x}$ and $F_{y}$, muscle forces and the foot orientation angles $\alpha$ and $\gamma$. Predicted peak and average pressures values and calculated percentage changes are listed in Tables 5, 6, 7, 8, 9, 10 and 11. Both the peak and average pressures increased with increased vertical GRF $F_{y}$ in all the ten regions. Larger percentage increases in peak pressures were observed at the heel-medial, heel-lateral, metatarsal 2 and 3, and toe 2–5 regions. The largest percentage increase occurred at the toe 2–5 region, which was $+$ 22.58% associated with 10% increase in $F_{y}$. The increased horizontal GRF $F_{x}$ (a braking force) resulted in increased peak and average pressures in the heel-medial, heel-lateral and mid-foot regions, but decreased plantar pressures in all the other seven forefoot regions. The peak pressures at metatarsals 2 and 3 and toe 2–5 regions were more sensitive to the $F_{x}$ change. A maximum $+$ 19.35% percentage change was found at the toe 2–5 region when $F_{x}$ decreased 10%. The maximum region-specific percentage change ratio was 1.9 for $F_{x}$ and 1.788 for $F_{y}$ in heel-medial and heel-lateral regions, and 1.904 for $F_{x}$ and 1.745 for $F_{y}$ in the mid-foot region. The maximum change ratio was 1.818 for $F_{x}$ and 1.818 for $F_{y}$ in the meta 1 and meta 2 regions, 1.528 for $F_{x}$ and 1.882 for $F_{y}$ in the meta 3, meta 4 and meta 5 regions, and 2.58 for $F_{x}$ and 2.58 for $F_{y}$ in the toe 1 and toe 2–5 regions.

Varying the three ankle plantar flexor muscle forces (LG, MG and SOL) produced very similar changes in peak and average plantar pressures (Fig. 7 and Tables 7, 8 and 9). When the muscle forces increased, peak and average pressures decreased in the heel-medial, heel-lateral and mid-foot regions, but increased in all the other seven regions. The metatarsal 2 and toe 2–5 regions showed higher sensitivity in response to changes in muscle forces than the other regions. The LG muscle had the least effect, and SOL muscle showed the largest effect on the plantar pressure changes in those two regions. A maximum 27.27% peak pressure change occurred at the metatarsal 2 region when SOL muscle force increased 10%. The maximum region-specific percentage change ratio for the three muscles was 1.23 for the heel-medial and heel-lateral regions, 2.063 for the mid-foot region, 2.727 for the meta 1 and meta 2 regions, 1.648 for the meta 3, meta 4 and meta 5 regions, and 3.226 for the toe 1 and toe 2–5 regions.

**Table 5:** Simulated peak and average plantar pressures (MPa) and their percentage changes (%) with respect to (w.r.t.) the baseline values in response to the change in vertical ground reaction force $F_{y}$ in ten plantar regions

Plantar region	Vertical ground reaction force $F_{y}$ *in N (percentage change w.r.t. baseline)*				
	349.33 ($-$ 10%)	368.75 ($-$ 5%)	388.15 (Baseline)	407.56 ($+$ 5%)	426.97 ($+$ 10%)
*Peak plantar pressure in MPa (percentage change % w.r.t. baseline)*					
Heel-medial	0.208 ($-$ 4.15)	0.212 ($-$ 2.30)	0.217	0.230 ($+$ 5.99)	0.249 ($+$ 14.75)
Heel-lateral	0.165 ($-$ 7.82)	0.171 ($-$ 4.47)	0.179	0.195 ($+$ 8.94)	0.206 ($+$ 15.08)
Mid-foot	0.052 ($-$ 17.46)	0.058 ($-$ 7.93)	0.063	0.065 ($+$ 3.17)	0.072 ($+$ 14.28)
Meta 5	0.078 ($-$ 8.23)	0.081 ($-$ 4.71)	0.085	0.093 ($+$ 9.41)	0.098 ($+$ 15.29)
Meta 4	0.059 ($-$ 18.06)	0.067 ($-$ 6.94)	0.072	0.074 ($+$ 2.78)	0.076 ($+$ 5.56)
Meta 3	0.050 ($-$ 5.66)	0.051 ($-$ 3.78)	0.053	0.057 ($+$ 7.55)	0.062 ($+$ 16.98)
Meta 2	0.041 ($-$ 6.82)	0.043 ($-$ 2.27)	0.044	0.048 ($+$ 9.09)	0.051 ($+$ 15.91)
Meta 1	0.038 ($-$ 7.32)	0.039 ($-$ 4.88)	0.041	0.043 ($+$ 4.87)	0.045 ($+$ 9.76)
Toe 2–5	0.025 ($-$ 19.35)	0.029 ($-$ 6.45)	0.031	0.035 ($+$ 12.90)	0.038 ($+$ 22.58)
Toe 1	0.047 ($-$ 9.62)	0.051 ($-$ 1.92)	0.052	0.057 ($+$ 9.61)	0.059 ($+$ 13.46)
*Average plantar pressure in MPa (percentage change % w.r.t. baseline)*					
Heel-medial	0.179 ($-$ 2.19)	0.182 ($-$ 0.55)	0.183	0.189 ($+$ 3.51)	0.197 ($+$ 7.65)
Heel-lateral	0.132 ($-$ 4.35)	0.136 ($-$ 1.45)	0.138	0.146 ($+$ 5.95)	0.151 ($+$ 9.42)
Mid-foot	0.049 ($-$ 7.55)	0.051 ($-$ 3.77)	0.053	0.054 ($+$ 1.89)	0.056 ($+$ 5.66)
Meta 5	0.060 ($-$ 7.69)	0.063 ($-$ 3.08)	0.065	0.069 ($+$ 6.15)	0.071 ($+$ 9.23)
Meta 4	0.057 ($-$ 8.06)	0.059 ($-$ 4.84)	0.062	0.063 ($+$ 1.61)	0.065 ($+$ 4.84)
Meta 3	0.030 ($-$ 6.25)	0.031 ($-$ 3.12)	0.032	0.034 ($+$ 6.25)	0.035 ($+$ 9.37)
Meta 2	0.029 ($-$ 9.37)	0.031 ($-$ 3.12)	0.032	0.035 ($+$ 9.37)	0.036 ($+$ 12.50)
Meta 1	0.032 ($-$ 3.03)	0.033 ($-$ 0.01)	0.033	0.034 ($+$ 3.03)	0.035 ($+$ 6.06)
Toe 2–5	0.019 ($-$ 9.52)	0.020 ($-$ 4.76)	0.021	0.022 ($+$ 4.76)	0.024 ($+$ 14.28)
Toe 1	0.029 ($-$ 9.37)	0.031 ($-$ 3.12)	0.032	0.034 ($+$ 6.25)	0.036 ($+$ 12.50)

**Table 6:** Simulated peak and average plantar pressures (MPa) and their percentage changes (%) with respect to (w.r.t.) the baseline values in response to the change in horizontal ground reaction force $F_{x}$ in ten plantar regions

Plantar region	Horizontal braking ground reaction force $F_{x}$ *in N (percentage change w.r.t. baseline)*				
	11.80 ($-$ 10%)	12.45 ($-$ 5%)	13.11 (baseline)	13.76 ($+$ 5%)	14.42 ($+$ 10%)
*Peak plantar pressure in MPa (percentage change % w.r.t. baseline)*					
Heel-medial	0.214 ($-$ 1.38)	0.215 ($-$ 0.92)	0.217	0.219 ($+$ 0.92)	0.220 ($+$ 1.38)
Heel-lateral	0.175 ($-$ 2.23)	0.176 ($-$ 1.68)	0.179	0.196 ($+$ 9.50)	0.205 ($+$ 14.52)
Mid-foot	0.059 ($-$ 6.35)	0.062 ($-$ 1.59)	0.063	0.069 ($+$ 9.52)	0.074 ($+$ 17.46)
Meta 5	0.093 ($+$ 9.41)	0.089 ($+$ 4.71)	0.085	0.084 ($-$ 1.18)	0.082 ($-$ 3.53)
Meta 4	0.074 ($+$ 2.78)	0.073 ($+$ 1.39)	0.072	0.066 ($-$ 8.33)	0.061 ($-$ 15.28)
Meta 3	0.059 ($+$ 11.32)	0.055 ($+$ 3.77)	0.053	0.051 ($-$ 3.77)	0.048 ($-$ 9.43)
Meta 2	0.051 ($+$ 15.91)	0.048 ($+$ 9.09)	0.044	0.042 ($-$ 4.55)	0.041 ($-$ 6.82)
Meta 1	0.042 ($+$ 2.44)	0.041 ($+$ 0.01)	0.041	0.041 ($-$ 0.01)	0.040 ($-$ 2.44)
Toe 2–5	0.037 ($+$ 19.35)	0.035 ($+$ 12.90)	0.031	0.029 ($-$ 6.45)	0.026 ($-$ 16.13)
Toe 1	0.056 ($+$ 7.69)	0.053 ($+$ 1.92)	0.052	0.052 ($-$ 0.01)	0.051 ($-$ 1.92)
*Average plantar pressure in MPa (percentage change % w.r.t. baseline)*					
Heel-medial	0.182 ($-$ 0.55)	0.183 ($-$ 0.01)	0.183	0.184 ($+$ 0.55)	0.185 ($+$ 1.09)
Heel-lateral	0.135 ($-$ 2.17)	0.136 ($-$ 1.45)	0.138	0.141 ($+$ 2.17)	0.146 ($+$ 5.80)
Mid-foot	0.050 ($-$ 5.66)	0.052 ($-$ 1.89)	0.053	0.055 ($+$ 3.77)	0.058 ($+$ 9.43)
Meta 5	0.070 ($+$ 7.69)	0.067 ($+$ 3.08)	0.065	0.064 ($-$ 1.54)	0.063 ($-$ 3.08)
Meta 4	0.063 ($+$ 1.61)	0.062 ($+$ 0.01)	0.062	0.060 ($-$ 3.23)	0.057 ($-$ 8.06)
Meta 3	0.034 ($+$ 6.25)	0.033 ($+$ 3.12)	0.032	0.031 ($-$ 3.12)	0.030 ($-$ 6.25)
Meta 2	0.035 ($+$ 9.37)	0.034 ($+$ 6.25)	0.032	0.031 ($-$ 3.12)	0.029 ($-$ 9.37)
Meta 1	0.034 ($+$ 3.03)	0.033 ($+$ 0.01)	0.033	0.033 ($-$ 0.01)	0.032 ($-$ 3.03)
Toe 2–5	0.023 ($+$ 9.52)	0.022 ($+$ 4.76)	0.021	0.020 ($-$ 4.76)	0.019 ($-$ 9.52)
Toe 1	0.034 ($+$ 6.25)	0.033 ($+$ 3.12)	0.032	0.032 ($-$ 0.01)	0.031 ($-$ 3.12)

**Table 7:** Simulated peak and average plantar pressures (MPa) and their percentage changes (%) with respect to (w.r.t.) the baseline values in response to the change in lateral gastrocnemius muscle force in ten plantar regions

Plantar region	Lateral gastrocnemius muscle force in N (percentage change w.r.t. baseline)				
	131.24 ($-$ 10%)	138.53 ($-$ 5%)	145.82 (baseline)	153.11 ($+$ 5%)	160.40 ($+$ 10%)
*Peak plantar pressure in MPa (percentage change % w.r.t. baseline)*					
Heel-medial	0.218 ($+$ 0.46)	0.217 ($+$ 0.01)	0.217	0.216 ($-$ 0.46)	0.215 ($-$ 0.92)
Heel-lateral	0.183 ($+$ 2.34)	0.181 ($+$ 1.12)	0.179	0.177 ($-$ 1.12)	0.174 ($-$ 2.79)
Mid-foot	0.068 ($+$ 11.47)	0.065 ($+$ 3.17)	0.063	0.062 ($-$ 1.59)	0.061 ($-$ 3.17)
Meta 5	0.082 ($-$ 3.53)	0.084 ($-$ 1.18)	0.085	0.086 ($+$ 1.18)	0.090 ($+$ 5.88)
Meta 4	0.061 ($-$ 15.28)	0.068 ($-$ 5.88)	0.072	0.072 ($+$ 0.01)	0.073 ($+$ 1.39)
Meta 3	0.051 ($-$ 3.77)	0.052 ($-$ 1.89)	0.053	0.055 ($+$ 3.77)	0.058 ($+$ 9.43)
Meta 2	0.042 ($-$ 4.55)	0.044 ($-$ 0.01)	0.044	0.045 ($+$ 2.72)	0.048 ($+$ 9.09)
Meta 1	0.040 ($-$ 2.44)	0.040 ($-$ 2.44)	0.041	0.041 ($+$ 0.01)	0.042 ($+$ 2.50)
Toe 2–5	0.029 ($-$ 6.45)	0.030 ($-$ 3.23)	0.031	0.032 ($+$ 3.22)	0.034 ($+$ 9.68)
Toe 1	0.051 ($-$ 1.92)	0.052 ($-$ 0.01)	0.052	0.052 ($+$ 0.01)	0.054 ($+$ 3.85)
*Average plantar pressure in MPa (percentage change % w.r.t. baseline)*					
Heel-medial	0.184 ($+$ 0.55)	0.183 ($+$ 0.01)	0.183	0.182 ($-$ 0.55)	0.181 ($-$ 1.09)
Heel-lateral	0.141 ($+$ 2.17)	0.139($+$ 0.72)	0.138	0.137 ($-$ 0.72)	0.136 ($-$ 1.45)
Mid-foot	0.056 ($+$ 5.66)	0.054 ($+$ 1.89)	0.053	0.052 ($-$ 1.89)	0.051 ($-$ 3.77)
Meta 5	0.062 ($-$ 4.62)	0.064 ($-$ 1.54)	0.065	0.066 ($+$ 1.54)	0.067 ($+$ 3.08)
Meta 4	0.059 ($-$ 4.84)	0.060 ($-$ 3.23)	0.062	0.062 ($+$ 0.01)	0.063 ($+$ 1.61)
Meta 3	0.031 ($-$ 3.12)	0.032 ($-$ 0.01)	0.032	0.034 ($+$ 6.25)	0.035 ($+$ 9.37)
Meta 2	0.030 ($-$ 6.25)	0.031 ($-$ 3.12)	0.032	0.033 ($+$ 3.12)	0.035 ($+$ 9.37)
Meta 1	0.032 ($-$ 3.03)	0.032 ($-$ 3.03)	0.033	0.033 ($+$ 0.01)	0.034 ($+$ 3.03)
Toe 2–5	0.020 ($-$ 4.76)	0.021 ($-$ 0.01)	0.021	0.022 ($+$ 4.76)	0.023 ($+$ 9.52)
Toe 1	0.031 ($-$ 3.12)	0.032 ($-$ 0.01)	0.032	0.032 ($+$ 0.01)	0.033 ($+$ 3.12)

**Table 8:** Simulated peak and average plantar pressures (MPa) and their percentage changes (%) with respect to (w.r.t.) the baseline values in response to the change in Medial Gastrocnemius muscle force in ten plantar regions

Plantar region	Medial gastrocnemius muscle force in N (percentage change w.r.t. baseline)				
	533.11 ($-$ 10%)	562.73 ($-$ 5%)	592.35 (baseline)	621.97 ($+$ 5%)	651.58 ($+$ 10%)
*Peak plantar pressure in MPa (percentage change % w.r.t. baseline)*					
Heel-medial	0.222 ($+$ 2.30)	0.219 ($+$ 0.92)	0.217	0.213 ($-$ 1.84)	0.211 ($-$ 2.77)
Heel-lateral	0.201 ($+$ 12.29)	0.189 ($+$ 5.59)	0.179	0.178 ($-$ 0.56)	0.175 ($-$ 2.23)
Mid-foot	0.076 ($+$ 20.63)	0.067 ($+$ 6.35)	0.063	0.060 ($-$ 4.76)	0.058 ($-$ 7.94)
Meta 5	0.075 ($-$ 11.76)	0.082 ($-$ 3.53)	0.085	0.092 ($+$ 8.24)	0.096 ($+$ 12.94)
Meta 4	0.062 ($-$ 13.89)	0.069 ($-$ 4.17)	0.072	0.073 ($+$ 1.39)	0.074 ($+$ 2.78)
Meta 3	0.052 ($-$ 1.89)	0.053 ($-$ 0.01)	0.053	0.054 ($+$ 1.89)	0.056 ($+$ 5.66)
Meta 2	0.041 ($-$ 6.82)	0.042 ($-$ 4.54)	0.044	0.049 ($+$ 11.36)	0.053 ($+$ 20.45)
Meta 1	0.038 ($-$ 7.32)	0.039 ($-$ 4.88)	0.041	0.042 ($+$ 2.44)	0.043 ($+$ 4.88)
Toe 2–5	0.030 ($-$ 3.33)	0.031 ($-$ 0.01)	0.031	0.035($+$ 12.90)	0.037 ($+$ 19.35)
Toe 1	0.049 ($-$ 5.77)	0.051 ($-$ 1.92)	0.052	0.053 ($+$ 1.92)	0.056 ($+$ 7.69)
*Average plantar pressure in MPa (percentage change % w.r.t. baseline)*					
Heel-medial	0.185 ($+$ 1.09)	0.184 ($+$ 0.55)	0.183	0.181 ($-$ 1.09)	0.179 ($-$ 2.18)
Heel-lateral	0.145 ($+$ 5.07)	0.141 ($+$ 2.17)	0.138	0.137 ($-$ 0.72)	0.135 ($-$ 2.17)
Mid-foot	0.058 ($+$ 9.43)	0.055 ($+$ 3.77)	0.053	0.051 ($-$ 3.77)	0.050 ($-$ 5.66)
Meta 5	0.060 ($-$ 7.69)	0.064 ($-$ 1.54)	0.065	0.068 ($+$ 4.61)	0.069 ($+$ 6.15)
Meta 4	0.057 ($-$ 8.06)	0.059 ($-$ 4.84)	0.062	0.062 ($+$ 0.01)	0.063 ($+$ 1.61)
Meta 3	0.031 ($-$ 3.12)	0.032 ($-$ 0.01)	0.032	0.032 ($+$ 0.01)	0.033 ($+$ 3.12)
Meta 2	0.030 ($-$ 6.25)	0.031 ($-$ 3.12)	0.032	0.034 ($+$ 6.25)	0.036 ($+$ 12.5)
Meta 1	0.031 ($-$ 6.06)	0.032 ($-$ 3.03)	0.033	0.033 ($+$ 0.01)	0.034 ($+$ 3.03)
Toe 2–5	0.021 ($-$ 0.01)	0.021 ($-$ 0.01)	0.021	0.021 ($+$ 0.01)	0.023 ($+$ 9.52)
Toe 1	0.031 ($-$ 3.12)	0.032 ($-$ 0.01)	0.032	0.032 ($+$ 0.01)	0.034 ($+$ 6.25)

**Table 9:** Simulated peak and average plantar pressures (MPa) and their percentage changes (%) with respect to (w.r.t.) the baseline values in response to the change in soleus muscle force in ten plantar regions

Plantar region	Soleus muscle force in N (percentage change w.r.t. baseline)				
	699.11 ($-$ 10%)	737.95 ($-$ 5%)	776.79 (baseline)	815.63 ($+$ 5%)	854.47 ($+$ 10%)
*Peak plantar pressure in MPa (percentage change % w.r.t. baseline)*					
Heel-medial	0.225 ($+$ 3.69)	0.221 ($+$ 1.84)	0.217	0.208 ($-$ 4.15)	0.202 ($-$ 6.91)
Heel-lateral	0.198 ($+$ 10.61)	0.188 ($+$ 5.03)	0.179	0.168 ($-$ 6.15)	0.160 ($-$ 10.61)
Mid-foot	0.073 ($+$ 15.87)	0.066 ($+$ 4.76)	0.063	0.061 ($-$ 3.17)	0.057 ($-$ 9.52)
Meta 5	0.079 ($-$ 7.06)	0.082 ($-$ 3.53)	0.085	0.092 ($+$ 8.24)	0.097 ($+$ 14.12)
Meta 4	0.069 ($-$ 4.17)	0.071 ($-$ 1.39)	0.072	0.072 ($+$ 0.01)	0.074 ($+$ 2.78)
Meta 3	0.048 ($-$ 9.43)	0.051 ($-$ 3.77)	0.053	0.054 ($+$ 1.89)	0.055 ($+$ 3.77)
Meta 2	0.039 ($-$ 11.36)	0.042 ($-$ 4.55)	0.044	0.048 ($+$ 9.09)	0.056 ($+$ 27.27)
Meta 1	0.035 ($-$ 14.63)	0.039 ($-$ 4.88)	0.041	0.043 ($+$ 4.88)	0.046 ($+$ 12.19)
Toe 2–5	0.027 ($-$ 12.90)	0.029 ($-$ 6.45)	0.031	0.035 ($+$ 16.13)	0.039 ($+$ 25.81)
Toe 1	0.050 ($-$ 3.85)	0.051 ($-$ 1.92)	0.052	0.054 ($+$ 3.85)	0.058 ($+$ 11.54)
*Average plantar pressure in MPa (percentage change % w.r.t. baseline)*					
Heel-medial	0.188 ($+$ 2.73)	0.185 ($+$ 1.09)	0.183	0.180 ($-$ 1.64)	0.177 ($-$ 3.28)
Heel-lateral	0.148 ($+$ 7.25)	0.142 ($+$ 2.17)	0.138	0.135 ($-$ 2.17)	0.129 ($-$ 6.52)
Mid-foot	0.059 ($+$ 11.32)	0.055 ($+$ 3.77)	0.053	0.052 ($-$ 1.89)	0.050 ($-$ 5.66)
Meta 5	0.061 ($-$ 6.15)	0.063 ($-$ 3.08)	0.065	0.068 ($+$ 6.15)	0.070 ($+$ 7.69)
Meta 4	0.061 ($-$ 1.61)	0.062 ($-$ 0.01)	0.062	0.062 ($+$ 0.01)	0.063 ($+$ 1.61)
Meta 3	0.030 ($-$ 6.25)	0.031 ($-$ 3.12)	0.032	0.032 ($+$ 0.01)	0.033 ($+$ 3.12)
Meta 2	0.029 ($-$ 9.37)	0.031 ($-$ 3.12)	0.032	0.034 ($+$ 6.25)	0.036 ($+$ 12.5)
Meta 1	0.030 ($-$ 9.09)	0.032 ($-$ 3.03)	0.033	0.033 ($+$ 0.01)	0.035 ($+$ 6.06)
Toe 2–5	0.019 ($-$ 9.52)	0.020 ($-$ 4.76)	0.021	0.022 ($+$ 4.76)	0.024 ($+$ 14.28)
Toe 1	0.031 ($-$ 3.12)	0.032 ($-$ 0.01)	0.032	0.033 ($+$ 3.12)	0.035 ($+$ 9.37)

Distinct changes in the peak and average plantar pressures occurred when foot orientation angles $\gamma$ and $\alpha$ changed (see Fig. 7 and Tables 10 and 11). Increases in $\gamma$ angle (equivalent to ankle dorsiflexion) led to increased peak and average pressures in the heel-medial and heel-lateral regions, but decreased pressures in all the other eight regions, and vice versa. Increases in $\alpha$ angle (equivalent to ankle eversion) resulted in increases in peak and average pressures in the heel-lateral, mid-foot, metatarsal 3, 4 and 5 regions, but decreased pressures in all other regions (and vice versa for decreases in $\alpha$). The metatarsal 1, 2 and 3, and toe 2–5 regions were more sensitive to changes in foot angle than the other areas. Changes in $\gamma$ angle had a larger effect on both peak and average pressures than the $\alpha$ angle. The two largest percentage changes were for the peak plantar pressures at the metatarsal 2 ($-$ 22.73%) and toe 2–5 region (19.35%), associated with 2 and $-$ 2% changes in $\gamma$ angle, respectively. The maximum region-specific percentage change ratio for $\alpha$ and $\gamma$ angle was 2.795 for the heel-medial and heel-lateral regions, 3.17 for the mid-foot region, 11.365 for the meta 1 and meta 2 regions, 9.43 for the meta 3, meta 4 and meta 5 regions, and 12.9 for the toe 1 and toe 2–5 regions.

**Table 10:** Simulated peak and average plantar pressures (MPa) and their percentage changes (%) with respect to (w.r.t.) the baseline values in response to the change in foot orientation angle $\upgamma$ in ten plantar regions

Plantar region	Foot orientation angle $\upgamma$ in degree (percentage change w.r.t. baseline)				
	$-$ 7.29 ($-$ 2%)	$-$ 7.22 ($-$ 1%)	$-$ 7.15 (baseline)	$-$ 7.08 ($+$ 1%)	$-$ 7.00 ($+$ 2%)
*Peak plantar pressure in MPa (percentage change % w.r.t. baseline)*					
Heel-medial	0.211 ($-$ 2.76)	0.215 ($-$ 0.92)	0.217	0.220 ($+$ 1.38)	0.222 ($+$ 2.30)
Heel-lateral	0.176 ($-$ 1.68)	0.177 ($-$ 1.12)	0.179	0.181 ($+$ 1.12)	0.189 ($+$ 5.59)
Mid-foot	0.064 ($+$ 1.59)	0.063 (0.01)	0.063	0.061($-$ 3.17)	0.060 ($-$ 4.76)
Meta 5	0.087 ($+$ 2.35)	0.086 ($+$ 1.18)	0.085	0.083 ($-$ 2.35)	0.079 ($-$ 7.06)
Meta 4	0.073 ($+$ 1.39)	0.072 (0.01)	0.072	0.071 ($-$ 1.39)	0.070 ($-$ 2.78)
Meta 3	0.058 ($+$ 9.43)	0.055 ($+$ 3.77)	0.053	0.048 ($-$ 9.43)	0.046 ($-$ 13.21)
Meta 2	0.046 ($+$ 4.55)	0.045 ($+$ 2.72)	0.044	0.041 ($-$ 6.82)	0.034 ($-$ 22.73)
Meta 1	0.042 ($+$ 2.44)	0.042 ($+$ 2.44)	0.041	0.040 ($-$ 2.44)	0.039 ($-$ 4.88)
Toe 2–5	0.037 ($+$ 19.35)	0.035 ($+$ 12.90)	0.031	0.030 ($-$ 3.23)	0.028 ($-$ 9.68)
Toe 1	0.053 ($+$ 1.92)	0.052 ($+$ 0.01)	0.052	0.051 ($-$ 1.92)	0.049 ($-$ 5.77)
*Average plantar pressure in MPa (percentage change % w.r.t. baseline)*					
Heel-medial	0.180 ($-$ 1.64)	0.182 ($-$ 0.55)	0.183	0.184 ($+$ 0.55)	0.185 ($+$ 1.09)
Heel-lateral	0.136 ($-$ 1.45)	0.137 ($-$ 0.72)	0.138	0.139 ($+$ 0.72)	0.142 ($+$ 2.90)
Mid-foot	0.054 ($+$ 1.89)	0.053 ($+$ 0.01)	0.053	0.052 ($-$ 1.89)	0.051 ($-$ 3.77)
Meta 5	0.066 ($+$ 1.54)	0.066 ($+$ 1.54)	0.065	0.064 ($-$ 1.54)	0.062 ($-$ 4.61)
Meta 4	0.063 ($+$ 1.61)	0.062 ($+$ 0.01)	0.062	0.062 ($-$ 0.01)	0.061 ($-$ 1.61)
Meta 3	0.035 ($+$ 9.37)	0.033 ($+$ 3.12)	0.032	0.031 ($-$ 3.12)	0.030 ($-$ 6.25)
Meta 2	0.033 ($+$ 3.12)	0.033 ($+$ 3.12)	0.032	0.031 ($-$ 3.12)	0.029 ($-$ 9.37)
Meta 1	0.034 ($+$ 3.03)	0.033 ($+$ 0.01)	0.033	0.032 ($-$ 3.03)	0.032 ($-$ 3.03)
Toe 2–5	0.023 ($+$ 9.52)	0.022 ($+$ 4.76)	0.021	0.021 ($-$ 0.01)	0.020 ($-$ 4.76)
Toe 1	0.032 ($+$ 0.01)	0.032 ($+$ 0.01)	0.032	0.032 ($-$ 0.01)	0.031 ($-$ 3.12)

**Table 11:** Simulated peak and average plantar pressures (MPa) and their percentage changes (%) with respect to (w.r.t.) the baseline values in response to the change in foot orientation angle $\upalpha$ in ten plantar regions

Plantar region	Foot orientation angle $\alpha$ in degree (percentage change w.r.t. baseline)				
	9.51 ($-$ 2%)	9.60 ($-$ 1%)	9.70 (baseline)	9.79 ($+$ 1%)	9.89 ($+$ 2%)
*Peak plantar pressure in MPa (percentage change % w.r.t. baseline)*					
Heel-medial	0.222 ($+$ 2.30)	0.218 ($+$ 0.46)	0.217	0.216 ($-$ 0.46)	0.215 ($-$ 0.92)
Heel-lateral	0.176 ($-$ 1.68)	0.177 ($-$ 1.12)	0.179	0.181 ($+$ 1.12)	0.184 ($+$ 2.79)
Mid-foot	0.062 ($-$ 1.59)	0.063 ($-$ 0.01)	0.063	0.064 ($+$ 1.59)	0.066 ($+$ 4.76)
Meta 5	0.082 ($-$ 3.53)	0.084 ($-$ 1.18)	0.085	0.086 ($+$ 1.18)	0.089 ($+$ 4.71)
Meta 4	0.070 ($-$ 2.78)	0.071 ($-$ 1.39)	0.072	0.072 ($+$ 0.01)	0.074 ($+$ 2.78)
Meta 3	0.052 ($-$ 1.89)	0.053 ($-$ 0.01)	0.053	0.054 ($+$ 1.89)	0.054 ($+$ 1.89)
Meta 2	0.048 ($+$ 9.09)	0.045 ($+$ 2.72)	0.044	0.044 ($-$ 0.01)	0.042 ($-$ 4.76)
Meta 1	0.044 ($+$ 7.32)	0.042 ($+$ 2.44)	0.041	0.040 ($-$ 2.44)	0.038 ($-$ 7.32)
Toe 2–5	0.034 ($+$ 9.68)	0.032 ($+$ 3.23)	0.031	0.030 ($-$ 3.23)	0.029 ($-$ 6.45)
Toe 1	0.054 ($+$ 3.85)	0.053 ($+$ 1.92)	0.052	0.052 ($-$ 0.01)	0.051 ($-$ 1.92)
*Average plantar pressure in MPa (percentage change % w.r.t. baseline)*					
Heel-medial	0.184 ($+$ 1.09)	0.183 ($+$ 0.01)	0.183	0.183 ($-$ 0.01)	0.182 ($-$ 0.55)
Heel-lateral	0.136 ($-$ 1.45)	0.136 ($-$ 1.45)	0.138	0.139 ($+$ 0.72)	0.141 ($+$ 2.17)
Mid-foot	0.052 ($-$1.89)	0.053 ($-$ 0.01)	0.053	0.053 ($+$ 0.01)	0.055 ($+$ 3.77)
Meta 5	0.064 ($-$ 1.54)	0.065 ($-$ 0.01)	0.065	0.065 ($+$ 0.01)	0.066 ($+$ 1.54)
Meta 4	0.061 ($-$ 1.61)	0.062 ($-$ 0.01)	0.062	0.062 ($+$ 0.01)	0.063 ($+$ 1.61)
Meta 3	0.031 ($-$ 3.12)	0.032 ($-$ 0.01)	0.032	0.032 ($+$ 0.01)	0.032 ($+$ 0.01)
Meta 2	0.033 ($+$ 3.12)	0.032 ($+$ 0.01)	0.032	0.032 ($-$ 0.01)	0.031 ($-$ 3.12)
Meta 1	0.032 ($+$ 3.03)	0.033 ($+$ 0.01)	0.033	0.033 ($-$ 0.01)	0.032 ($-$ 3.03)
Toe 2–5	0.020 ($+$ 4.76)	0.021 ($+$ 0.01)	0.021	0.021 ($-$ 0.01)	0.022 ($-$ 4.76)
Toe 1	0.033 ($+$ 3.12)	0.032 ($+$ 0.01)	0.032	0.032 ($-$ 0.01)	0.032 ($-$ 0.01)

## Discussion

FE foot models supplement experimental studies to assess the complex biomechanical behaviour of the foot and predict the unmeasurable internal stress and strain states. The FE model in this study comprises subject-specific ankle–foot geometry; muscle loading (provided by a subject-specific multi-body musculoskeletal model); and boundary conditions (defined by subject-specific 3D GRFs and 3D foot orientation angles). In contrast, most previous studies estimated muscle forces either from literature data (Chen et al. 2010; Cheung et al. 2005; Guiotto et al. 2014; Wong et al. 2015) or based on assumptions that muscle force is linearly proportional to PCSA (Chen et al. 2012) or muscle EMG signal (Bae et al. 2015). Moreover, the vertical GRF component (Guiotto et al. 2014; Bae et al. 2015) or the sagittal plane position of the ankle–foot (Chen et al. 2012) has been used to define boundary conditions.

This study modelled all the major musculoskeletal components in the foot complex. The relative articulating movements of all the bony joints were modelled using 74 cartilage layers for 37 pairs of articulations between 30 bony structures. Furthermore, to better represent the physiological constraints of the ligamentous structures, 85 ligament bundles consisting of 1814 truss elements and a 3D solid bulk plantar fascia were constructed in the model. This contrasts with previous work that simplified the plantar fascia to linear or curved line elements (Chen et al. 2010; Bae et al. 2015; Brilakis et al. 2012; Isvilanonda et al. 2012). The modelling strategy used in this study enables the entire ankle–foot musculoskeletal structure to self-adapt to static equilibrium configurations in response to external loading and boundary conditions. Moreover, a model that allows physiological joint articulations could be used to assess joint kinematics, kinetics and joint contacts within the foot musculoskeletal complex (Chen et al. 2012).

The predicted region-specific peak and average plantar pressures are in good agreement with the measured barefoot walking pressures of the same subject in all ten plantar regions and at all five events in walking. All the percentage errors for both peak and average pressures were < 10% with the exception of the average pressure in the heel-lateral region at early stance. This is perhaps due to the intensified plantar pressure in the heel area, since in early stance all load passes through this subsection of the heel. The gait measurement data confirm that foot position, plantar pressure, ground reaction and muscle forces change significantly during gait (see Tables 2, 3 and Fig. 6). Achieving the accuracy reported during such variable conditions suggests the detailed FE construction and subject-specific modelling strategy used in this study have represented the complex biomechanical behaviour of the human foot reasonably well.

Sensitivity studies were conducted to investigate the effects of key modelling parameters and also probe the self-adaptive behaviour of the foot musculoskeletal structure. The peak and average plantar pressures increased with hardening plantar soft tissue and vice versa with softening tissue. Plantar pressures tended to concentrate under metatarsals 2 and 3 and toes 2–5 regions with increased plantar soft tissue stiffness. This agrees with the simulation results of (Cheung et al. 2005) and is consistent with clinical observations that plantar foot ulcers are frequently found at the medial forefoot at sites of hardened skin calluses (Mueller et al. 1994; Raspovic et al. 2000). The percentage rate of change in the peak plantar pressure with hardener soft tissues was generally higher than that in previous FE studies (Cheung et al. 2005), probably because linear elastic material rather than hyperplastic materials was used in this study.

As expected, increased vertical GRF $F_{y}$ led to increased plantar pressure in all the plantar regions, with more intensified pressures at the heel and the second and third metatarsal areas. Interestingly, increases in horizontal braking GRF $F_{x}$ resulted in plantar pressure increases in the rear foot whilst pressure decreased in the forefoot. This suggests horizontal GRF could cause load transfer between the rear and forefoot. In the most sensitive medial forefoot region (toe 2–5), 10% increase in braking $F_{y}$ lead to a 19.35% increase in peak plantar pressure. This strongly suggests that both vertical and horizontal GRF need to be considered in FE foot modelling. This also implies that to understand elevated plantar pressures and their associated foot problems, horizontal braking and accelerating GRF are important in addition to the vertical GRF.

Increases in force from the three ankle plantar flexor muscles LG, MG and SOL all generated similar changes in plantar pressure with decreased peak pressures in the heel regions and increased forefoot pressures. This is plausible because increased ankle plantar flexor muscle forces have a tendency to lift the heel off the ground and hence transfer plantar pressures from the heel to the forefoot. However, despite sharing the same muscle/tendon insertion site at the upper ridge of the calcaneus, different plantar flexor muscles produced different changes in the region-specific peak pressures. The maximum region-specific percentage change ratio (pressure percentage change over muscle force percentage change) was 1.528 for LG, 2.063 for MG and 2.727 for SOL. This is very likely due to the distinct force magnitudes and 3D force vector directions of the three muscles, suggesting that the muscle force data provided by the multi-body musculoskeletal models have a large impact on the FE simulations. Moreover, different ankle flexor muscles at the same insertion site may have different contributions to plantar loads (Chen et al. 2012).

The FE foot model predicted the responses of the foot musculoskeletal structure to the changes in foot orientation angles well. With increased ankle dorsiflexion, plantar pressures increased in the heel regions and decreased in all forefoot regions. When the ankle dorsiflexes, the calcaneus moves towards the ground surface whilst the forefoot moves away from the ground. Similarly, with increased ankle inversion, the plantar pressures increased in all the lateral regions and reduced in all medial regions. The maximum region-specific percentage change ratio (pressure percentage change over orientation angle percentage change) was 11.37 for angle $\gamma$, and 4.84 for angle $\alpha$, which are much larger than those of the other model parameters. This strongly suggests that the 3D rather than 2D positions and orientations of the foot structure needs to be carefully defined. Subject-specific measurement data might be a prerequisite for this in future FE foot modelling. This also suggests the model would be responsive to known changes in foot biomechanics after injury, such as changes in ankle inversion post-lateral ankle sprain (Bae et al. 2015).

The encapsulated soft tissue was simplified as linear elastic material to reduce the computational load incurred by the intensive FE sensitivity analyses. Nonlinear representation, e.g. hyperelastic material, was used to model the plantar soft tissue in previous studies (Chen et al. 2010; Cheung et al. 2005; Qian et al. 2013). However, in most previous work, the bones, cartilage, ligaments, tendons, plantar fascia etc. were all modelled as linear elastic materials based on data in the literature. Latest advances in medical imaging techniques provide in vivo personalised nonlinear nonhomogeneous material property data for FE foot modelling (Brandenburg et al. 2014; Passmore et al. 2017). Although muscle forces used in the FE simulations of this study were provided by a subject-specific multi-body musculoskeletal model (based on the 3D motion and GRF data recorded for the same subject), the accuracy of the calculated muscle forces needs careful verification and validation (Hicks et al. 2015). Direct measurement of in vivo muscle loads is required to truly validate musculoskeletal models, but it remains a significant challenge. Quasi-static FE simulations were conducted at five typical events in the stance phase of walking, and fully muscle-driven dynamic FE foot simulations may improve the prediction accuracy of peak plantar pressures (Qian et al. 2013). However, this is still challenging for 3D simulations due to the high computational demands and the complicated interactions between foot musculoskeletal components under dynamic conditions.

The FE prediction results were only validated against measured plantar pressure data in this study. Since the relative articulations of all the bony joints were included in the foot FE model, the predicted 3D joint motions and internal soft tissue deformations can also be experimentally validated (Chen et al. 2012). Our future work will look to address this limitation by comparing use of subject-specific model output to 3D in vivo foot joint motion data from dual-plane fluoroscopy and internal soft tissue deformations recorded by high-resolution MRI.

## Conclusion

A subject-specific FE foot model was constructed with boundary and loading conditions defined by a multi-body musculoskeletal model and 3D gait measurements for the same subject. The model provides good predictions of personalised barefoot walking plantar pressures and reasonable adaptive behaviours to changes in external loading and boundary conditions. The sensitivity analyses reveal the model predictions are moderately sensitive to material properties, GRFs and muscle forces, but significantly sensitive to foot orientation. The FE modelling approach presented provides a possible way to explore the sophisticated interplay between muscular control, internal joint movements, plantar load transfer and ground–foot interaction for the human foot.
